# Co‐Producing Peritoneal Dialysis Nursing Sensitive Indicators for Quality Care: A Multinational Consensus Building Design

**DOI:** 10.1111/jorc.70008

**Published:** 2025-01-28

**Authors:** Jessica Baillie, Ann Bonner, Sonia Guillouet, Cornelia Mikut, Sally Punzalan, Anna Kaczmarek, Lawrence Climaco Wenzyl, Jeanette Finderup

**Affiliations:** ^1^ School of Healthcare Sciences Cardiff University Cardiff UK; ^2^ School of Nursing and Midwifery Griffith University, Australia and Aarhus University Gold Coast Denmark; ^3^ Health Training and Research Centre University of Caen Caen France; ^4^ Home Dialysis Division KfH, Kuratorium für Dialyse und Nierentransplantation Neu‐Isenburg Germany; ^5^ Renal Home Therapies Imperial College Healthcare NHS Trust London UK; ^6^ Medical Affairs, Baxter Zurich Switzerland; ^7^ Peritoneal Dialysis AKH University Hospital Vienna Vienna Austria; ^8^ Department of Renal Medicine Aarhus University Hospital Aarhus Denmark

**Keywords:** consensus, nursing, peritoneal dialysis, quality care

## Abstract

**Background:**

Nursing sensitive indicators are a way of measuring aspects of patient care that are most affected by the actions of the nurse. Despite the existence of nursing sensitive indicators, these are largely not suitable to measure peritoneal dialysis nursing practice.

**Objective:**

This project aimed to co‐develop a set of peritoneal dialysis nursing‐sensitive indicators.

**Design:**

Informed by the Donabedian quality framework (structure, process, outcome), a multinational co‐production consensus design was used.

**Participants and Measurements:**

First, an expert panel of seven professionals proposed potential indicators from clinical expertise and examining peer‐reviewed articles and clinical guidelines. Next, the expert panel undertook a consensus building process involving face‐to‐face meetings and online discussion to refine the indicators. Lastly indicator confirmation was undertaken using a 5‐point rating scale involving delegates at a major conference.

**Results:**

The initial indicator proposal, based on evidence and clinical experience, identified 65 potential indicators (20 structural, 22 process and 23 outcome). The consensus process involved discussion and negotiation to reduce the potential indicators to 28 (eight structural, 12 process and eight outcome). Confirmation involved 25 nurses with all 28 indicators supported (all > 3.5/5). Indicators highly supported were patient satisfaction, fluid balance assessment, peritoneal dialysis catheter exit‐site, clinical signs measurement, peritonitis investigation, peritoneal dialysis catheter complications referral and infection rates.

**Conclusion:**

Following further validity, reliability and feasibility testing, these nursing sensitive indicators can be used to measure the quality of peritoneal dialysis nursing care provided for patients and families.

## 1 Introduction

Peritoneal dialysis (PD) is an important treatment option for kidney failure, used globally by approximately 11% of patients (Cho et al. 2021). This patient group have complex healthcare needs and require expert, high quality care. Centre effect is a key factor in PD technique survival (Guillouët et al. 2016), which could relate to greater clinical competence and experience of healthcare professionals in centres with greater PD experience (Bello et al. 2022). Nurses have a vital role in educating patients to live with PD (Nopsopon et al. 2022), supporting individuals and their families to develop their knowledge, skills and abilities to safely use this home‐based treatment (Figueiredo et al. 2016).

## 2 Literature Review

The Donabedian (1988) framework is frequently used to understand the quality of care within a health service. This framework identifies the structural (attributes of the setting, including material resources, human resources and organisational structure), process (practices of giving and receiving healthcare), and outcome (impact of healthcare on patients) indicators of quality, and is widely utilised to examine quality of nursing care (McCullough et al. 2023; Mustonen et al. 2024; Oner et al. 2020). Within kidney care, this framework has been utilised to provide examples of quality indicators (van der Veer et al. 2014), including nurse‐to‐patient ratio (structural), hepatitis B vaccination for patients using haemodialysis (process), and satisfaction with kidney care (outcome). Despite 40 indicators being used across Canada to measure the quality of home dialysis (Dubrofsky et al. 2020), four indicators (home dialysis prevalence, home dialysis incidence, rates of PD‐associated peritonitis, and home dialysis attrition) were most used by the provinces, although there was little consistency.

Nursing sensitive indicators (NSI) are used in many countries to measure the quality of nursing care (Heslop, Lu, and Xu 2014). A recent systematic review of 39 studies from 1997 to 2017 identified the most frequently reported NSI, which included nurse staffing, mortality, and nosocomial infections (Oner et al. 2020). Using an adapted Donabedian framework, structural, process, patient‐focused outcomes and nursing‐focused outcomes NSI were identified, although the authors highlighted a lack of standardisation and consistency of NSI definitions across the literature. Several studies have identified haemodialysis NSI (Chen et al. 2023; Gao et al. 2018; McIntyre, Coyer, and Bonner 2020). There was some consistency between these indicators, for instance nurse‐to‐patient ratio (Chen et al. 2023; Gao et al. 2018; McIntyre, Coyer, and Bonner 2020), while two studies identified patient satisfaction as an outcome indicator (Chen et al. 2023; McIntyre, Coyer, and Bonner 2020). However, variation was notable, for example routine foot assessment for patients utilising haemodialysis was only identified as a NSI in one study (McIntyre, Coyer, and Bonner 2020). There is thus variation in the NSI proposed for haemodialysis nursing.

Indicators to measure the quality of PD nursing practice are lacking and suitable indicators need to encompass nursing support for patients with complex care needs across clinical settings (i.e., hospital, community and home). The aim of this project was to co‐develop PD NSI.

## 3 Materials and Methods

### 3.1 Study Design

**Figure 1:**
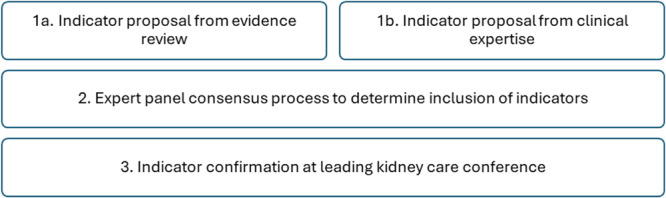
Peritoneal dialysis nursing sensitive indicators co‐development process.

**Figure 2:**
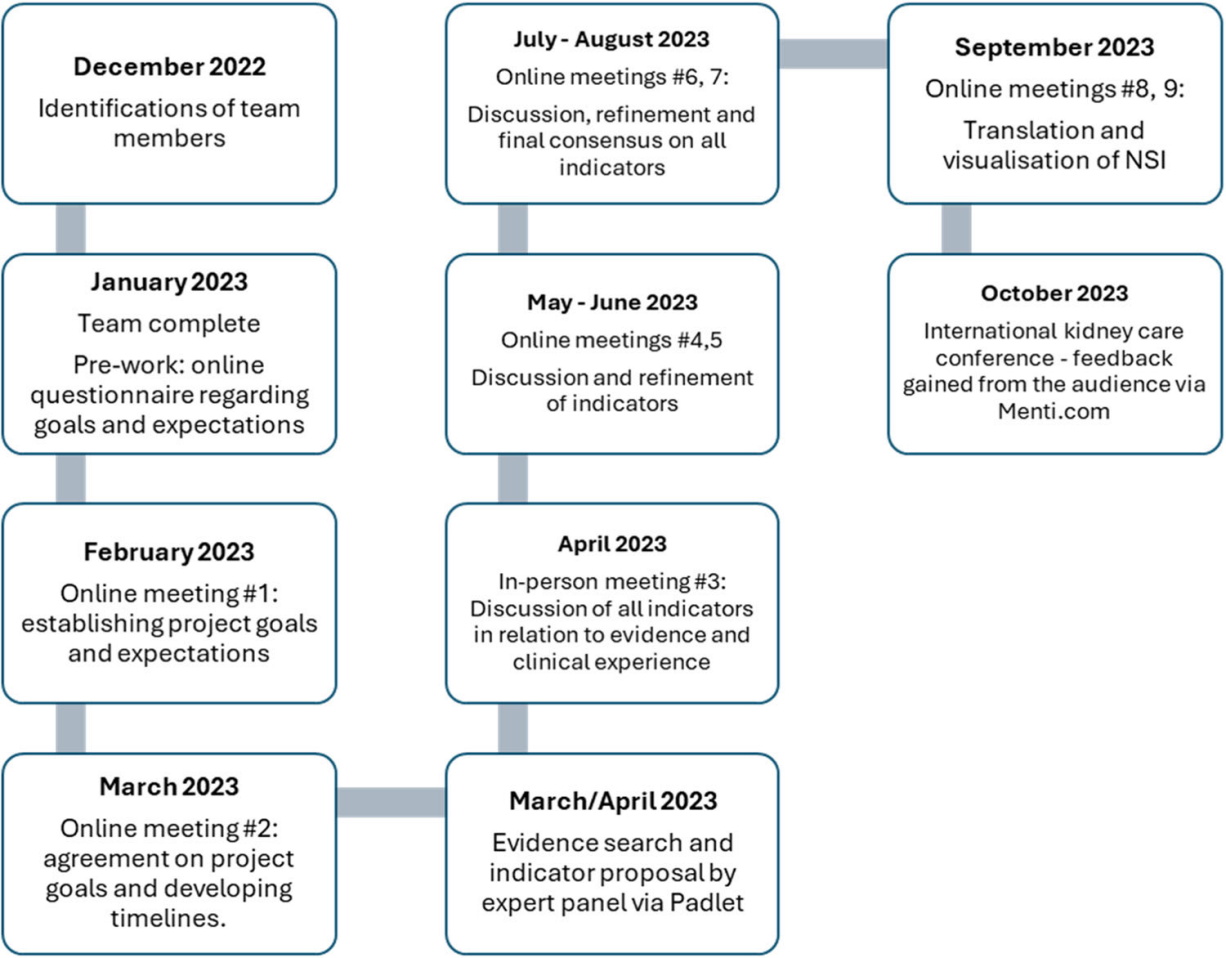
Project overview.

This project used a multinational, co‐production, consensus building design involving a panel working collaboratively. The panel was assembled by the clinical academic lead and industry lead to represent different European countries: United Kingdom, Denmark, Germany, Austria, France and Switzerland. The panel had expertise in PD nursing practice and research (mean average 17.5 years) and members were working in clinical practice (*n* = 3), academia (*n* = 2) and in clinical academic roles (*n* = 2). Four panel members had PhDs and one member had a Masters degree. The project involved three stages: indicator proposal (from professional experience and evidence review); expert consensus; and indicator confirmation by kidney care professionals (Figure 1). The process embedded the evidence‐based practice tripartite: evidence; skills, knowledge, experiences and judgment of professionals; and input from stakeholders who may rely on the resources provided (Eldredge 2024). This project utilised a co‐production, consensus building process and did not collect personal data, therefore ethical approval was not required. The process began in January 2023 and the set of indicators were presented for confirmation in October 2023 (Figure 2).

### 3.2 Stage One: Indicator Proposal

The panel firstly defined the PD NSI as a set of criteria and a way of measuring aspects of patient and family care that are most effected by the actions of the PD nurse. Using the Donabedian framework (Donabedian 1988), PD NSI were conceptualised as structural (organisation and distribution of PD nursing services), process (interactions between PD nurses, patients and their families); and outcome (effects of PD nursing care). The expert panel then proposed potential structural, process and outcome indicators, which were identified from reviewing evidence and the panel's professional expertise. This was an iterative process that involved the expert panel reflecting on the relevance of evidence considering their professional expertise and searching for evidence that supplemented clinical knowledge. This interactive and collaborative process was supported by online meetings and an online software program (Padlet.com).

Evidence was identified through a search of Ovid Embase/MEDLINE, CINAHL, Elsevier's Science Direct and Clarivate's Web of Science in April 2023, undertaken by the expert panel and an information specialist from Baxter. Initially the search terms were related to synonyms of NSI and quality indicators, and peritoneal dialysis; Boolean operators and truncation were used to enhance the sensitivity and specificity of the search, and no language or date restrictions were applied, but no relevant articles were identified. The search terms were broadened to remove the reference to peritoneal dialysis specifically and 43 publications were identified. Reference lists were checked to find additional studies. Clinical guidelines were identified from the National Institute for Health and Care Excellence, International Society for Peritoneal Dialysis (ISPD) and European Dialysis and Transplant Nurses Association/European Renal Care Association (EDTNA/ERCA). Final articles and guidelines were reviewed by two of the panel (JB and JF) to ensure these met the inclusion/exclusion criteria: a peer‐reviewed article or clinical guideline, focus on healthcare quality, and relevant to kidney care.

### 3.3 Stage Two: Expert Panel Consensus to Determine Indicator Inclusion

Following the evidence review, the expert panel reviewed the PD NSI in a collaborative, consensus building process. Collaboration requires effective communication, partnership, equal opportunities amongst members and respect (Bansal et al. 2019). Clear communication was facilitated through regular meetings and the use of the online collaborative software (Padlet.com). The approach adopted principles of the Delphi method, characterised by rounds of reviewing the NSI with the opportunity for reflection and feedback on the results of the previous round, to develop a consensus view on the PD NSI (Barrett and Heale 2020).

The potential indicators were discussed at a face‐to‐face meeting, which involved exploring the research evidence for each indicator, sharing professional perspectives, and reflecting on whether the indicator measured: the quality of PD nursing care and the quality of structures, processes or outcomes of PD nursing care. The face‐to‐face meeting was followed by a further four online meetings where indicators were discussed, collapsed, removed and refined. The final consensus process used an online survey where panel members voted on whether an indicator should be included or excluded, and whether it was structural/process/outcome indicator. Rather than setting a percentage for consensus (van Zuuren et al. 2022), the expert panel negotiated to ensure all members agreed with the final indicators.

### 3.4 Stage Three: Indicator Confirmation

The final stage was indicator confirmation at a leading European kidney care conference with delegates who would eventually utilise the PD NSI. First, the process of co‐developing the indicators was outlined and the PD NSI were presented in turn to conference delegates. The delegates were then asked to rate their level of agreement of including each NSI in the final selection, using online software (Menti.com). Delegates rated each indicator using a Likert scale, with 0 *strongly disagreeing* with the inclusion of the PD NSI, and 5 *strongly agreeing* with the inclusion of the PD NSI, similar to previous research on NSI (McIntyre, Coyer, and Bonner 2020). Rating was chosen, rather than ranking, as this allowed the panel to examine how far apart the indicators were in terms of appreciation (Del Grande and Kaczorowski 2023). The mean rating for each indicator was automatically generated for delegates to view, with discussion about each indicator facilitated by the panel.

## 4 Results

### 4.1 Stage One: Indicator Proposal

The final literature search identified eight relevant articles that included potential indicators. This was supplemented with four clinical guidelines, and a wider range of peer‐reviewed articles identified by the expert panel. Following review of the evidence and reflection on clinical expertise, a total of 65 PD NSI were identified. There were 20 structural indicators covering nurse‐to‐patient ratio, PD nurse education, and acute PD provision; 22 process indicators encompassing a range of patient assessment processes, patient and family PD training, and shared decision making; and 23 outcome indicators related to satisfaction, technique survival, and percentage of staff with a post‐registration qualification.

### 4.2 Stage Two: Expert Panel Consensus

During this stage, the 65 PD NSI were firstly reduced to a potential set of 45 indicators: 14 structural, 15 process and 16 outcome. The final 28 PD NSI were grouped into: eight structural indicators to measure how PD nursing services are organised and distributed, including qualification and number of PD nurses; 12 process indicators to measure the direct nursing care provided to patients on PD and their families; and eight outcome indicators that measure the effects of PD nursing care on patients and their families as well as the job satisfaction of PD nurses. All members of the panel agreed with the inclusion, categorisation and phrasing of each indicator.

### 4.3 Stage Three: Indicator Confirmation

25 nurses participated in the indicator confirmation via attendance at a conference workshop which had delegates from 44 countries. All 28 indicators were supported (all scoring > 3.5/5). Structural indicators highly supported were PD nurse training programme, shared decision‐making programme, PD training programme and home visit programme (range 4–4.3). Regarding process indicators, fluid balance assessment achieved the highest score of 4.8/5, while care plan development and implementation was rated the lowest at 4.2/5. Seven of the eight outcome indicators achieved mean confirmation scores > 4.3/5, with patient satisfaction achieving (4.9/5) and infection rates (4.7/5). The full list of indicators, with a description of the scope of the indicator, supporting literature and conference mean confirmation score, is provided in Table 1.

**Table 1:** Overview of PD Nursing Sensitive Indicators.

	Indicator	Description	Supporting literature	Mean confirmation score/5
Structural	PD nurse training programme	Existence of a PD nurse training programme	Oner et al. (2020)	4.3
	Nurse‐to‐patient ratio	Appropriate PD nurse‐to‐patient ratio	Chen et al. (2023); Gao et al. (2018); Machado de Oliveria et al. (2020); McIntyre, Coyer and Bonner (2020); van der Veer et al. (2014)	3.7
	Shared decision‐making programme^a^	Existence of a shared decision‐making programme for treatment planning and PD prescription	National Institute for Health and Care Excellence (2018)	4.2
	PD training programme^a^	Existence of an evidence‐based PD training programme	Figueiredo (2022); Figueiredo et al. (2016)	4.2
	Acute PD nursing pathway^a^	Provision of an acute PD nursing pathway	Cullis et al. (2021); Ghaffari, Kumar and Guest (2013)	3.8
	Home visit programme^a^	Provision of a home visit programme	Coulthard and Pearce (2022); Figueiredo et al. (2016)	4
	Remote patient management^a^	Provision of a remote patient management programme (communication such as phone calls)	Figueiredo et al. (2016)	3.9
	Assisted PD^a^	Availability of assisted PD (e.g., older people, frail, learning disability, care giver respite)	Dubrofsky et al. (2020)	3.9
Process	Fluid balance assessment	Assessing the patient's fluid balance (ultrafiltration rate, hydration status, residual kidney function)	Branco (2022); McIntyre, Coyer and Bonner (2020)	4.8
	Sleep quality assessment^a^	Assessing the patient's sleep quality	Liu et al. (2021)	4.4
	Nutritional assessment^a^	Assessing the patient's nutritional status	Pecolar and Bricman (2022)	4.3
	Bowel habit assessment^a^	Assessing bowel habits and preventing constipation	Marques and Guedes (2022)	4.5
	PD exit‐site^a^	Assessing the PD catheter exit‐site	Marques and Guedes (2022)	4.6
	Clinical signs measurement	Measuring the patient's clinical signs (weight, blood pressure, pulse, oxygen saturation, blood glucose level (if diabetic), 24 h urine)	Branco (2022); McIntyre, Coyer and Bonner (2020)	4.6
	Documentation	Documenting the patient's progress accurately	McIntyre, Coyer and Bonner (2020)	4.5
	PD catheter complications referral^a^	Referring the patient for catheter complications promptly	Marques and Guedes (2022)	4.7
	Peritonitis investigation^a^	Investigating the cause of peritonitis	Marques and Guedes (2022)	4.7
	Care plan development and implementation	Developing and implementing person‐centred PD care plans (awareness of what to observe and how to react, including re‐training)	Heslop, Lu and Xu (2014)	4.2
	Self‐management support	Providing self‐management support for patients (to develop knowledge, skills and confidence)	Gliki (2022); Oner et al. (2020)	4.6
	Assisted PD education^a^	Educating home care nurses for assisted PD	Gliki (2022)	4.5
**Outcome**	PD incidence^a^	Incidence of PD	Dubrofsky et al. (2020)	4.4
	PD prescription adequacy^a^	Adequacy of PD prescription, including target weight control	Branco (2022); Gao et al. (2018)	4.4
	PD technique survival^a^	PD technique survival (1–3 year)	Dubrofsky et al. (2020)	4.6
	Infection rates	PD related infection rates (exit site infection, peritonitis, tunnel infection)	Gao et al. (2020; Gao et al. (2018); Li et al. (2022); Manera et al. (2020); McIntyre, Coyer and Bonner (2020)	4.7
	Nurse satisfaction	Nurse satisfaction levels	Heslop, Lu and Xu (2014); McIntyre, Coyer and Bonner (2020); Oner et al. (2020)	4.3
	Patient satisfaction	Patient satisfaction levels	Chen et al. (2023); McIntyre, Coyer and Bonner (2020); van der Veer et al. (2014)	4.9
	Caregiver satisfaction	Caregiver satisfaction levels	Heslop, Lu and Xu (2014)	4.3
	Nursing qualification	Percentage of nurses with post‐registration (kidney) nursing qualification	Aiken et al. (2014)	3.9

^a^ Specific for PD and not identified in other NSIs.

## 5 Discussion

This study reached a consensus among an expert panel of PD nurses on 28 PD NSIs to measure quality care. These included eight structural, 12 process and eight outcome indicators. All indictors were confirmed and supported by 25 nurses during the confirmation process. While 12 indicators have been identified in earlier studies of NSIs, 16 were specific to the practice of PD nurses.

### 5.1 Structural Indicators

In terms of human resources required in practice, a common NSI is the *nurse‐to‐patient ratio* (Oner et al. 2020). A study in Brazil recommended a ratio of approximately one nurse to 20‐25 patients, based on a 6–8 h working day (Figueiredo et al. 2016). PD nurses are required to have advanced knowledge of PD, including the ability to educate, monitor and supervise patients using this treatment (Schaepe and Bergjan 2015), which necessitates a comprehensive *training programme for the PD nurse*. The “Train the Trainer” programme for PD nurses covers clinical knowledge, principles of home dialysis, case studies, clinical competencies and assessment (J. S. F. Chow et al. 2019), although it requires evaluation.

The presence of a *shared decision‐making programme* for treatment planning and PD prescription is recommended as a structural indicator. However, shared decision‐making should be integrated not only into the treatment decision but also as a fundamental principle of treatment planning processes. PD care involves a partnership between the professional, patient, and the patient's family, with the aim of supporting the patient in actively participating in their own care (Gliki 2022). The development and use of an *evidence‐based PD training programme* is a critical factor for improving PD clinical outcomes (Figueiredo 2022). The ISPD syllabus for teaching PD to patients and their family members is based on adult learning principles, and can be tailored to individual learning needs (Figueiredo et al. 2016). A recent scoping review however highlights the variety of published PD training strategies and emphasises the need for further research in this area (Jaelani et al. 2023).

When providing PD nursing services, an *acute PD nursing pathway* is essential. This pathway requires staff to insert an abdominal catheter, support treatment and train patients quickly (Chan et al. 2019). Without an acute PD pathway, patients may face limited dialysis options (Woodrow et al. 2017). Home visits by PD nurses offers several benefits, including the assessment of the home environment, evaluation of patient technique, and providing psychosocial support (Zhang, Hawley, and Johnson 2016), although these home visits require a significant time commitment. In community settings, where therapy is supported by tertiary care, effective communication methods are crucial for ensuring safe treatment and patient well‐being (Figueiredo et al. 2016). This can be facilitated through a *remote patient management programme*. Software that collects and uploads treatment data for review by the PD centre has been associated with improved clinical outcomes (Yeter, Manani, and Ronco 2021). An *assisted PD programme* involves training community nurses, healthcare technicians or care home staff to perform PD for patients who cannot self‐manage and lack family support (Giuliani et al. 2017). A recent position statement from the ISPD highlights the health, psychosocial and economic benefits of assisted PD programmes, and notes the variability in assisted PD services provided globally (Oliver et al. 2024).

### 5.2 Process Indicators

PD nursing assessment of the patient encompasses multiple aspects. *Fluid balance assessment* indicates the patient's hydration status, as well as ultrafiltration rate and residual kidney function. Indeed, clinical guidelines for PD proficiency highlight the importance of identifying residual kidney function (Branco 2022), due to the impact on patient survival and quality of life. *Assessment of sleep* is identified as a middle tier core outcome for SONG‐PD (Standardised Outcomes in Nephrology, No date). A systematic review of qualitative studies exploring patients' experiences of sleep when receiving dialysis overnight, found that automated PD disrupted sleep due to alarms, noise and technical complications (Cheng et al. 2021). *Nutritional assessment* is important as patients on PD are at risk of malnutrition due to protein loss (Shao et al. 2024), thus requiring a protein intake of 1.2 g/kg/day, increasing during peritonitis (Kiebalo et al. 2020). While dieticians have a key role in the multidisciplinary kidney team, nurses involved in the practice of PD also make an important contribution (Pecolar and Bricman 2022). Nursing *assessment of the PD catheter exit site* includes erythema, induration or tenderness (Marques and Guedes 2022). Infection is a priority outcome identified by SONG‐PD (Standardised Outcomes in Nephrology, No date), and catheter‐related infections is a risk factor for peritonitis and catheter loss (K. M. Chow et al. 2023). For patients on PD, *assessing bowel habits* and treating constipations are important to maximise the function of the catheter (Marques and Guedes 2022). Constipation has been reported as a predictor for peritonitis in patients using continuous ambulatory PD (Su et al. 2012). Accurate *measurement of clinical signs* is crucial and includes tracking weight, blood pressure, pulse rate, oxygen saturation, blood glucose level (for patients with diabetes) and 24‐h urine volume (to assess residual kidney function). Accurate *documentation of the patient's progress* and nursing assessments is essential in all healthcare settings.

Complications related to the PD catheter include infection, malfunction, and contamination (Marques and Guedes 2022). While nursing processes can manage some of these complications, *timely referral of catheter complication* to other members of the clinical team is crucial for optimal patient outcomes. Unfortunately, peritonitis remains the most common treatment‐related complication of PD (Marshall 2022), making *investigating the cause of peritonitis* an important NSI. However, peritonitis can be distressing and stigmatising for patients and their families (Baillie, Gill, and Courtenay 2023), thus investigating its causes requires both skill and sensitivity to effectively reduce the risk of reinfection. Within PD, care planning is crucial for enhancing patients' treatment experiences and overall well‐being (Coulthard and Pearce 2022). *Person‐centred PD care plans* should address areas such as assessment, prevention, recognition, and management of complications. *Providing self‐management support* is essential to help patients effectively manage their PD, by building their knowledge, skills, and confidence. Supporting patients in self‐management is the primary objective of nurses (Gliki 2022). The ISPD syllabus for training nurses to teach patients about PD can also be adapted for *educating caregivers about assisted PD* (Figueiredo et al. 2016). Effective partnerships between the PD nurse, patient, patient's family and caregivers is fundamental for the success of PD (Gliki 2022).

### 5.3 Outcome Indicators

PD nurses have a crucial role in supporting patients as they being PD treatment through pre‐dialysis care, including providing evidence‐based PD training. Their expertise is essential for achieving the desired *incidence of PD* within their centres. In the UK, the Getting it Right First Time (GIRFT) Renal Medicine speciality report mandates a 20% prevalent rate for home dialysis (Lipkin and McKane 2021). This requires PD nurses to support patients initiating dialysis and to provide ongoing expert care. *Adequacy of PD prescription* is multifaceted and is defined as an “effective dose able to control symptoms, preserve patient's activity, and maintain sufficient metabolic and homoeostatic balance” (Branco 2022, p.29). ISPD practice recommendations emphasise the importance of shared decision‐making and using multiple assessments to ensure high quality PD care including patient reported outcome measures, fluid status, nutrition status, and toxin removal (Brown et al. 2020). As outlined in the process indicators, nursing assessments are vital for ensuring the effectiveness of PD prescriptions. *Technique survival* is a core outcome for SONG‐PD (Standardised Outcomes in Nephrology, No date), and the primary outcome of the large study on PD outcomes and practice patterns (P‐DOPPS) (Perl et al. 2016). The UK Kidney Association monitors 1 year PD‐technique survival in the UK Renal Registry annual report, and here we additionally recommended 3‐year survival as a PD NSI to recognise the impact of nursing care on peritonitis and constipation prevention. *PD infection* is another critical outcome for PD (Manera et al. 2020; Standardised Outcomes in Nephrology, No date), with the ISPD expecting centres to measure and report peritonitis rates annually, aiming for < 0.4 episodes per patient‐year (Li et al. 2022).

## 6 Strengths and Limitations

This project is subject to several limitations. Firstly, a systematic review was not conducted, although a systematic search was performed to identify pertinent evidence. The expert panel was comprised predominantly of members from Western European countries, resulting in a lack of representation from other regions, particularly developing countries. Nevertheless, the final phase of the project took place during a conference attended by delegates from a broader array of countries, who affirmed the relevance of the PD NSI. This inclusion may enhance the global applicability of these indicators, although future research should ensure inclusion of developing countries offering peritoneal dialysis.

## 7 Implications for Clinical Practice

Retention of nursing staff is a global challenge (Marques and Guedes 2022), including in kidney care (Boyle et al. 2022). Focusing on high quality care and acknowledging the contributions of staff are two strategies for reducing staff turnover (Marufu et al. 2021). Identifying and acknowledging high quality PD nursing care is therefore crucial. The utilisation of PD varies worldwide and is influenced by factors at the patient level (micro), the healthcare team level (meso), and the healthcare system level (macro). These PD NSI provide the opportunity for PD nurses to benchmark, promote and audit high‐quality care across these levels. At the micro level, these PD NSI can enhance nursing documentation practices. At the meso level, the indicators can support the development of competence development programme for PD nurses. Lastly, at a macro level, PD NSI can contribute to the creation of national or international quality registers.

**Figure 3:**
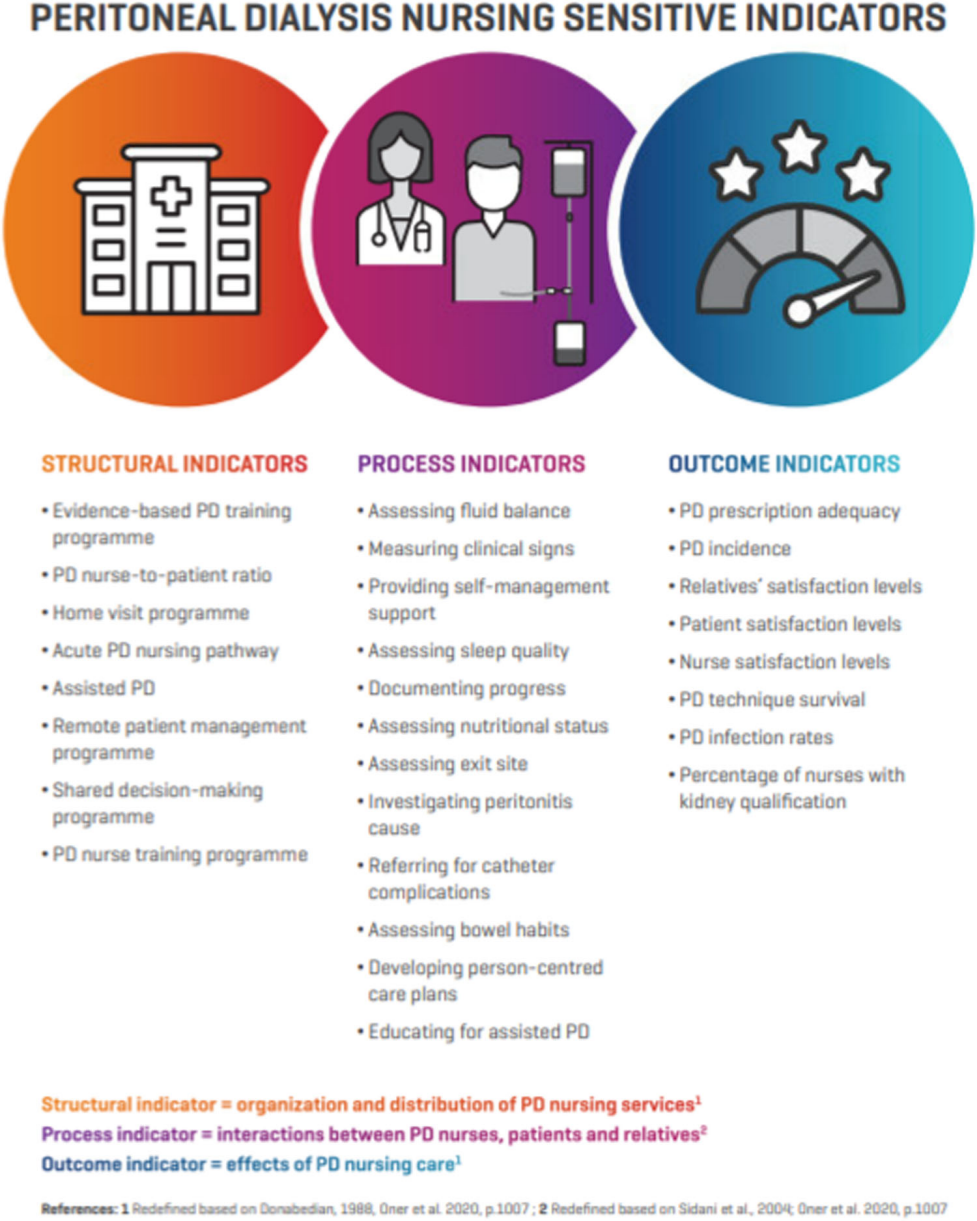
Image of the peritoneal dialysis nursing sensitive indicators.

To assist with the dissemination and implementation of the PD NSIs, an image of the indicators has been developed (Figure 3) and the indicators have been translated into nine different European languages besides English; Czech, Danish, French, German, Greek, Italian, Polish, Portuguese and Spanish. The PD NSIs have been endorsed by EDTNA/ERCA (https://www.edtnaerca.org/collaboration/industry-partners).

## 8 Conclusion

Assessing the quality of PD nursing practice is crucial to ensure that patients and families receive high standards of care. Until now, no indicators existed to measure the quality of PD nursing practice. This article has reported the first NSI specifically for PD, applicable to nursing care provided in hospitals, communities or patients' homes. These indicators now enable both internal and external benchmarking of PD nursing care. Further research is needed to develop an audit tool and then to evaluate the feasibility, validity and reliability of using these indicators.

## Author Contributions

All authors contributed to project design and execution, and drafting and agreeing the final manuscript.

## Conflicts of Interest

The authors declare no conflicts of interest.
